# Curcumin and synthetic analogs induce reactive oxygen species and decreases specificity protein (Sp) transcription factors by targeting microRNAs

**DOI:** 10.1186/1471-2407-12-564

**Published:** 2012-11-30

**Authors:** Shruti U Gandhy, KyoungHyun Kim, Lesley Larsen, Rhonda J Rosengren, Stephen Safe

**Affiliations:** 1 College of Medicine, Texas A&M Health Sciences Center, Houston, TX, 77030, USA; 2 Institute of Biosciences and Technology, Texas A&M Health Science Center, 2121 W. Holcombe Blvd, Houston, TX, 77030, USA; 3 Department of Chemistry, University of Otago, P.O. Box 56, Dunedin, 9054, New Zealand; 4 Department of Pharmacology and Toxicology, University of Otago, P.O. Box 913, Dunedin, 9054, New Zealand; 5 Department of Veterinary Physiology and Pharmacology, Texas A&M University, 4466 TAMU, College Station, TX, 77843-4466, USA

**Keywords:** Curcumin, ROS induction, Sp transcription factors, MicroRNAs

## Abstract

**Background:**

Curcumin inhibits growth of several cancer cell lines, and studies in this laboratory in bladder and pancreatic cancer cells show that curcumin downregulates specificity protein (Sp) transcription factors Sp1, Sp3 and Sp4 and pro-oncogenic Sp-regulated genes. In this study, we investigated the anticancer activity of curcumin and several synthetic cyclohexanone and piperidine analogs in colon cancer cells.

**Methods:**

The effects of curcumin and synthetic analogs on colon cancer cell proliferation and apoptosis were determined using standardized assays. The changes in Sp proteins and Sp-regulated gene products were analysed by western blots, and real time PCR was used to determine microRNA-27a (miR-27a), miR-20a, miR-17-5p and ZBTB10 and ZBTB4 mRNA expression.

**Results:**

The IC_50_ (half-maximal) values for growth inhibition (24 hr) of colon cancer cells by curcumin and synthetic cyclohexanone and piperidine analogs of curcumin varied from 10 μM for curcumin to 0.7 μM for the most active synthetic piperidine analog RL197, which was used along with curcumin as model agents in this study. Curcumin and RL197 inhibited RKO and SW480 colon cancer cell growth and induced apoptosis, and this was accompanied by downregulation of specificity protein (Sp) transcription factors Sp1, Sp3 and Sp4 and Sp-regulated genes including the epidermal growth factor receptor (EGFR), hepatocyte growth factor receptor (c-MET), survivin, bcl-2, cyclin D1 and NFκB (p65 and p50). Curcumin and RL197 also induced reactive oxygen species (ROS), and cotreatment with the antioxidant glutathione significantly attenuated curcumin- and RL197-induced growth inhibition and downregulation of Sp1, Sp3, Sp4 and Sp-regulated genes. The mechanism of curcumin-/RL197-induced repression of Sp transcription factors was ROS-dependent and due to induction of the Sp repressors ZBTB10 and ZBTB4 and downregulation of microRNAs (miR)-27a, miR-20a and miR-17-5p that regulate these repressors.

**Conclusions:**

These results identify a new and highly potent curcumin derivative and demonstrate that in cells where curcumin and RL197 induce ROS, an important underlying mechanism of action involves perturbation of miR-ZBTB10/ZBTB4, resulting in the induction of these repressors which downregulate Sp transcription factors and Sp-regulated genes.

## Background

Traditional medicines have been extensively used for treatment of multiple diseases including cancer, and many widely used anticancer drugs are derived from natural sources [1,2]. Curcumin is a major aromatic constituent of turmeric (*Curcuma longa*) and has been widely investigated for its anticancer activities in multiple cancer cell lines and *in vivo* tumor models [3,4]. Curcumin has been used in clinical trials for pancreatic cancer, and it is anticipated that curcumin or a suitable derivative will eventually play a clinical role in cancer chemotherapy as a “stand alone” drug or in combination therapies [5-9]. A major problem associated with the use of curcumin is its low bioavailability and this has resulted in efforts to improve formulations for delivery of curcumin and also to develop curcumin analogs that are more potent and more bioavailable [5,10-14].

The focus on curcumin as an anticancer agent is due, in part, to its broad spectrum of activities. Curcumin inhibits cancer cell and tumor growth, decreases survival, and inhibits angiogenesis and inflammation. Many, but not all of these responses, are observed in different cancer cell lines, and several pathways and genes responsible for these effects have been reported [3,4]. For example, curcumin-induced growth arrest and apoptosis in various HCT-116-derived colon cancer cells was due to induction of various caspases and inhibition of β-catenin signaling pathways [15]. Other studies in colon cancer cells report similar responses and also show downregulation of cyclin D1, bcl-2, VEGF and p65 (NFκB) and other pro-oncogenic factors [15-19].

Studies in this laboratory have shown that curcumin inhibits bladder and pancreatic cancer cell and tumor growth and that the anticancer activity is due, in part, to downregulation of specificity protein (Sp) transcription factors Sp1, Sp3, Sp4 and Sp-regulated genes [20,21]. Sp transcription factors are overexpressed in multiple cancer cell lines and tumors [20-26] and represent an example of non-oncogene addiction by cancer cells [27,28], and this is primarily due to the pro-oncogenic activity of Sp-regulated genes. Results of drug (including curcumin) treatment and Sp knockdown by RNA interference have identified Sp-regulated genes that are important for cell proliferation [cyclin D1, epidermal growth factor receptor (EGFR), hepatocyte growth factor receptor (c-MET)], survival (bcl-2, survivin), angiogenesis [vascular endothelial growth factor (VEGF) and its receptors (VEGR)], and inflammation (p65 and p50) [20-22,29-32]. In this study, we investigated the anticancer activities of curcumin and several synthetic analogs using colon cancer cells as a model. Our major objectives were to compare the relative potencies of curcumin with the synthetic analogs, to determine their effects on Sp transcription factors and Sp-regulated genes, and the mechanisms responsible for downregulation of Sp transcription factors.

Both curcumin and the most active synthetic analog RL197 inhibited colon cancer cell growth with an IC_50_ (growth inhibition) of 10 and 0.7 μM, respectively. Both compounds induced reactive oxygen species (ROS) and downregulated Sp1, Sp3, Sp4 and Sp-regulated genes, and these responses were attenuated by the antioxidant glutathione (GSH). The mechanism of curcumin-/RL197-induced repression of Sp transcription factors was ROS-dependent and due to induction of the Sp repressors ZBTB10 and ZBTB4 and downregulation of microRNAs (miR)-27a, miR-20a and miR-17-5p that regulate the repressors.

## Methods

### Cell lines, reagents and antibodies

RKO and SW480 human colon carcinoma cell lines and CCD-18Co colon fibroblasts were obtained from American Type Culture Collection (Manassas, VA). Cells were initially grown and multiple aliquots were frozen and stored at -80°C for future use. Cells were purchased more than 6 months ago and were not further tested or authenticated by the authors. Cells were maintained in Dulbecco’s modified Eagle’s Medium (DMEM) with phenol red supplemented with 10% FBS, and 10 mL/L of 100X antibiotic/antimycotic solution (Sigma-Aldrich Co., St. Louis, MO). Cells were cultured in 150-cm^2^ plates in an air/CO_2_ (95:5) atmosphere at 37°C. All antibodies were purchased from Santa Cruz Biotechnology (Santa Cruz, CA) except c-MET and survivin (Cell Signaling Technology, Danvers, MA), NFκB-p50 and NFκB-p65 (Abcam Inc., Cambridge, MA), Sp1 (Millipore, Billerica, MA), and FAS (Sigma-Aldrich Co., St. Louis, MO). Glutathione, 98% (γ-L-glutamyl-L-cysteinyl-glycine, GSH) and lactacystin (proteasome inhibitor) were purchased from Sigma-Aldrich. Carboxy-H_2_DCFDA was purchased from Invitrogen (Carlsbad, CA). Curcumin (98% pure) was purchased from Indofine Chemical Company, Inc. (Hillsborough, NJ), and curcumin analogs were synthesized as described [14] and RL197 synthesis is outlined below.

### Chemical synthesis

Melting points were determined on a Mettler Toledo FP62 melting block and were uncorrected. High resolution mass spectrometry was recorded using a VG70-250S double focusing magnetic sector mass spectrometer. NMR spectra, at 25°C, were recorded at 500 MHz for ^1^H and 125 MHz for ^13^C on Varian INOVA-500 spectrometer. Chemical shifts are given in ppm on the δ scale referenced to the solvent peaks C**H**Cl_3_ at 7.26 and **C**DCl_3_ at 77.00. 1-Boc-4-piperidone, and 2,5-dimethoxybenzaldehyde were purchased from the Sigma-Aldrich Company. **(3E,5E)-3,5-Bis(2,5-dimethoxybenzylidene)-1-t-butoxycarbonylpiperidin-4-one (RL197).** To a mixture of 1-Boc-4-piperidone (0.70 g, 3.5 mmol) and 2,5-dimethoxybenzaldehyde (1.20 g, 7.4 mmol) in methanol (50 mL) was added sodium methoxide (5M, 0.75 ml) and the mixture was stirred for 18 hr at room temperature. The resulting precipitate was removed by filtration, then washed with cold methanol and purified by recrystallisation from ethanol to give RL197 as a yellow solid (1.20 g, 69%); mp 167.7°C. Found: C, 67.79; H, 6.79; N, 2.73. C_28_H_33_NO_7_ requires: C, 67.86; H, 6.71; N, 2.83. ^1^H-NMR (CDCl_3_) δ: 1.26 (s, 9H), 3.79 (s, 6H), 3.82 (s, 6H), 4.59 (bs, 4H), 6.80 (bs, 2H), 6.85 (d, *J* = 9Hz, 2H), 6.89 (dd, *J* = 2, 9 Hz, 2H), 7.95 (bs, 2H); ^13^C-NMR (CDCl_3_) δ: 187.76, 154.35, 153.05, 152.74, 133.30 (br), 124.83, 116.13, 115.46 (br), 111.80, 80.20, 56.04, 55.84, 45.05, 28.05: (HRMS (+ve ESI) calc for C_28_H_33_NaNO_7_: 518.2149 *m*/*z* [MNa^+^], found: 518.2115 *m*/*z*.

### Cell proliferation assay and annexin V staining

RKO and SW480 cancer cells were seeded in DMEM High Glucose with 10% FBS on 12-well plates and allowed to attach for 24 hr. The medium was then changed to DMEM High Glucose containing 2.5% charcoal-stripped FBS and cells were treated with either the vehicle (DMSO) or the indicated compounds for 24 hr. Cells were trypsinized and counted using a Coulter Z1 particle counter. For Annexin V staining, cells were seeded in 6-well plates, allowed to attach overnight, and treated with curcumin or RL197 as indicated. Annexin V and propidium iodide staining was determined using the Vybrant apoptosis assay kit #2 (Molecular Probes, Grand Island, NY) and images were captured at 20X magnification using IN cell analyzer 6000 (GE Healthcare Biosciences, Piscataway, NJ).

### Western blots

RKO and SW480 cancer cells were seeded in DMEM High Glucose with 10% FBS on 6-well plates and allowed to attach for 24 hr. The medium was then changed to DMEM High Glucose containing 2.5% charcoal-stripped FBS and treated with either the vehicle (DMSO) or the indicated compounds and analyzed by western blots as described [20,21].

### ROS estimation

Cellular ROS levels were evaluated with the cell permeant probe carboxy-H_2_DCFDA (5-(and-6)-carboxy-2^′^,7^′^-dichlorodihydrofluorescein diacetate) from Invitrogen. Following treatment, cells seeded on 6-well plates were loaded with 10 mM of carboxy-H_2_DCFDA for 1 hr, washed once with serum-free medium, and analyzed for ROS levels using BD Accuri C6 Flow Cytometer using the FL1 channel. Analysis of data was determined with BD Accuri CFlow software (set at 480 nm and 525 nm excitation and emission wavelengths, respectively). Each experiment was carried out in triplicate and results are expressed as means ± S.E. for each treatment group.

### Measurement of mitochondrial membrane potential (MMP)

MMP was measured with Mitochondrial Membrane Potential Detection Kit (Stratagene, La Jolla, CA) according to the manufacturer’s protocol using the JC-1 dye; mitochondrial membrane potential was measured using BD Accuri C6 Flow Cytometer and data were analyzed using the BD Accuri CFlow software. J-aggregates are detected as red fluorescence and J-monomers are detected as green fluorescence. Each experiment was determined in triplicate, and results are expressed as means ± S.E. for each treatment group.

### Quantitative real time PCR of mRNA and miRNAs

The mirVana miRNA Isolation Kit (Applied Biosystems, Carlsbad, CA) was used for miRNA extraction and, miRNAs (RNU6B, miRNA-27a, miRNA-20a, and miRNA 17-5p) were quantitated by real time PCR using the Taqman miRNA assay (Applied Biosystems) according to the manufacturer’s protocol. U6 small nuclear RNA (RNU6B) was used as a control to determine relative miRNA expression. mRNA was extracted using the RNeasy Protect Mini kit (Qiagen, Valencia, CA) according to the manufacturer’s protocol, and cDNA was prepared by reverse transcription using Reverse Transcription Kit (Promega, Madison, WI) according to the manufacturer’s protocol. Each PCR was carried out in triplicate using SYBR Green PCR Master Mix (Invitrogen) at one cycle of 95°C for 10 min, 40 cycles of 95°C for 15 s, and 60°C for 1 min on MyIQ2 Real Time PCR Detection System (BioRad, Hercules, CA) with 1 μmol/L of each primer and 1 μL cDNA template in each 20 μL reaction. TATA binding protein (TBP) was used as an endogenous control to compare the relative mRNA levels. Comparative CT methods were used for relative quantitation of samples and the following primers, purchased from Integrated DNA Technologies (Coralville, IA ), were used:

Sp1 (Forward): 5^′^-TCA CCT GCG GGC ACA CTT-3^′^

Sp1 (Reverse): 5^′^-CCG AAC GTG TGA AGC GTT-3^′^

TBP (Forward): 5^′^-TGCACAGGAGCCAAGAGTGAA-3^′^

TBP (Reverse): 5^′^-CACATCACAGCTCCCCACCA-3^′^

ZBTB10 (Forward): 5^′^-GCTGGATAGTAGTTATGTTGC-3^′^

ZBTB10 (Reverse): 5^′^-CTGAGTGGTTTGATGGACAGA-3^′^

ZBTB4 (Forward): 5^′^-ACCTGTGCAGGAATTTCCAC-3^′^

ZBTB4 (Reverse): 5^′^-GAGCGGCCAAGTTACTGAAG-3^′^

Primers for Sp3 and Sp4 were purchased from Qiagen.

### Statistical analysis

Statistical significance of differences between the treatment groups was determined using the Student’s t test, and levels of probability were noted. IC_50_ values were calculated using linear regression analysis and expressed in micromolar (μM) concentrations at 95% confidence intervals.

## Results

### Curcumin inhibits colon cancer cell growth and downregulates Sp transcription factors and Sp-regulated genes

**Figure 1:**
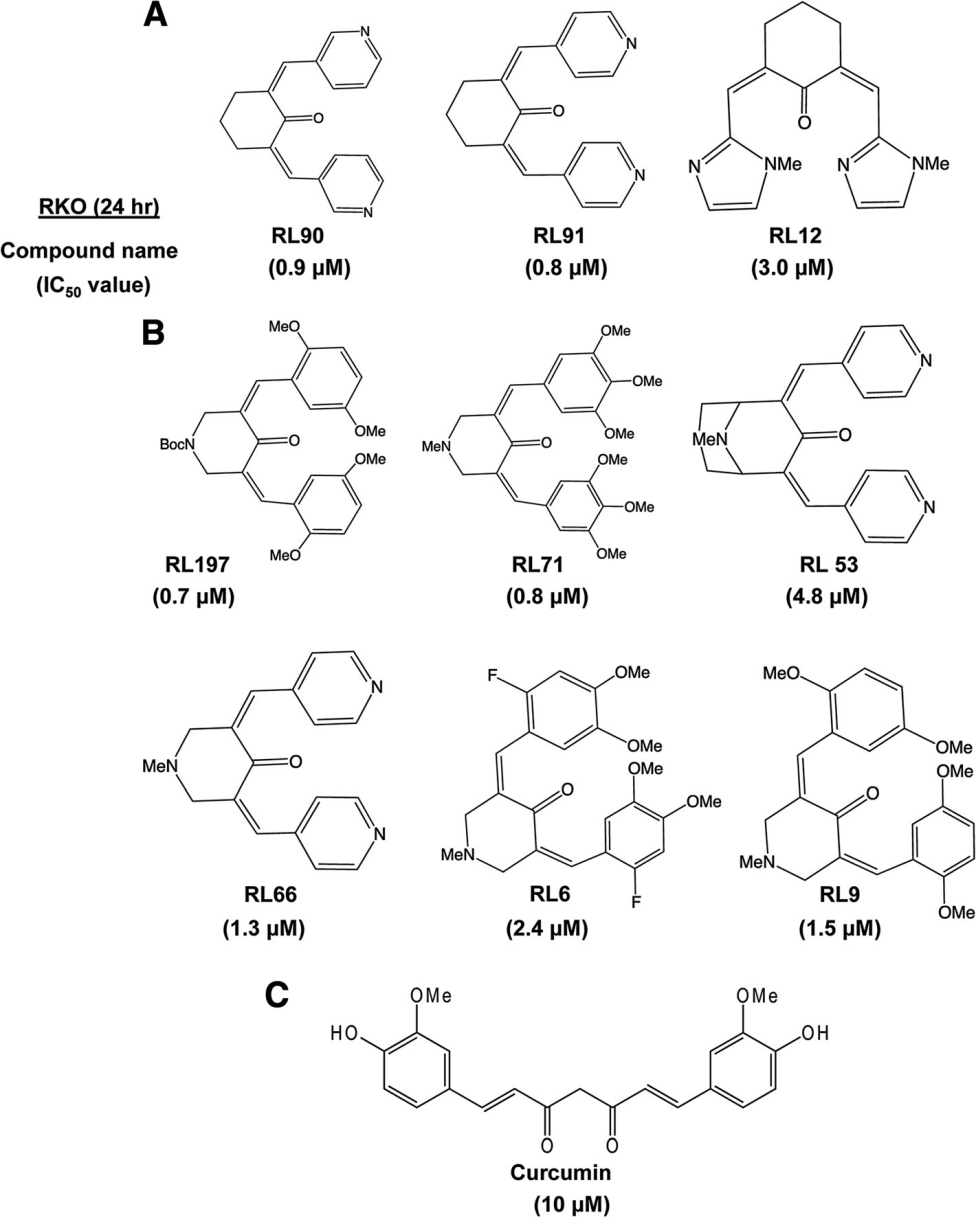
**Structures of cyclohexanone (A) and piperidone (B) analogs and curcumin (C) and their growth inhibitory IC**_**50 **_**values.** Growth inhibition was determined as outlined in the Materials and Methods.

**Figure 2:**
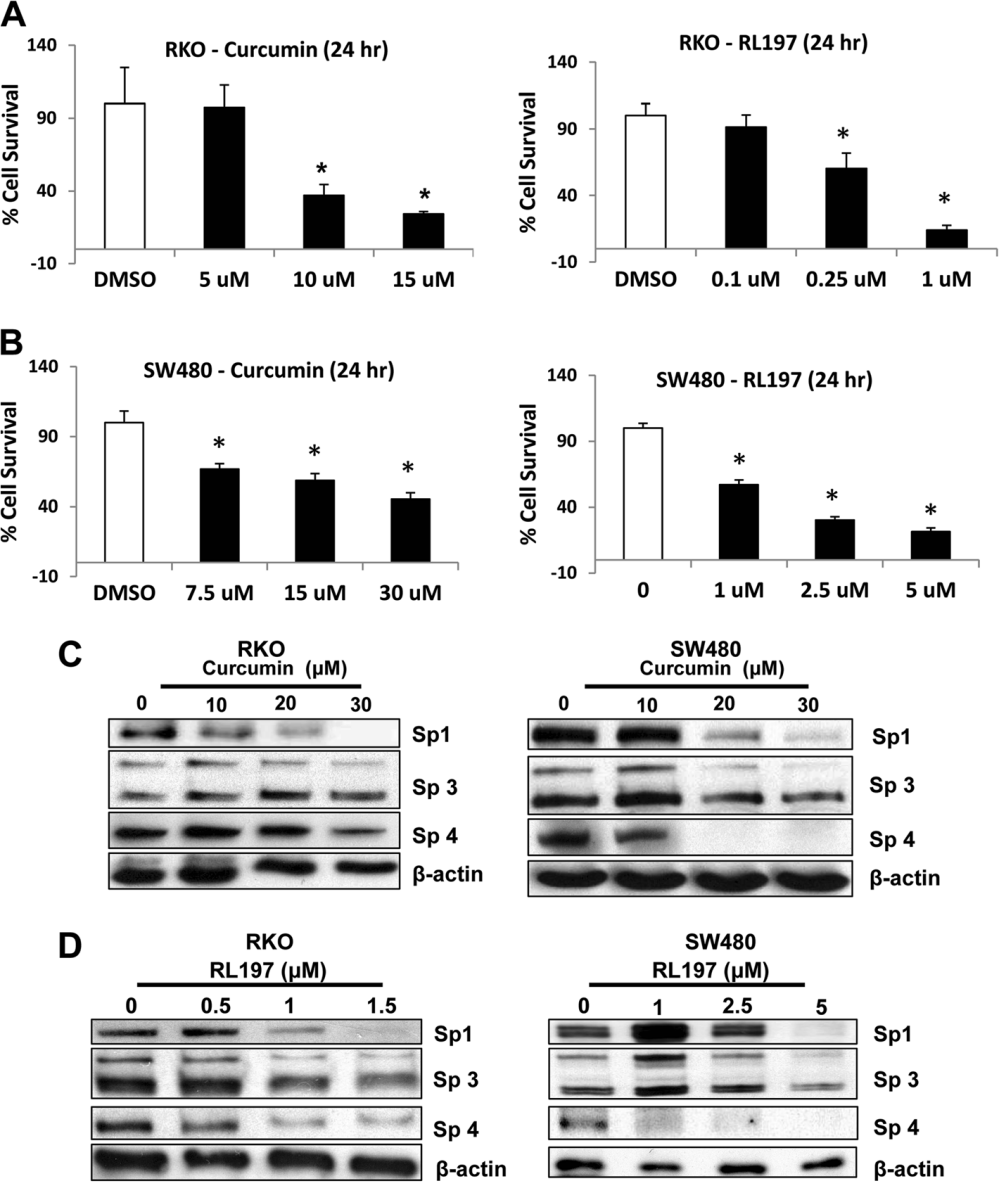
**Curcumin and RL197 decrease RKO and SW480 cell growth and downregulate Sp1, Sp3 and Sp4 proteins.** RKO (**A**) and SW480 (**B**) cells were treated with DMSO or different concentrations of curcumin and RL197, and after 24 hr, cell growth was determined as outlined in the Materials and Methods. Results are means ± S.E. for at least 3 replicate determinations and significant (p < 0.05) inhibition compared to DMSO (set at 100%) is indicated (*). Downregulation of Sp1, Sp3 and Sp4 proteins by different concentrations of curcumin (**C**) and RL197 (**D**) (after 24 hr) in RKO and SW480 cells was determined by western blot analysis of whole cell lysates as described in the Materials and Methods.

**Figure 3:**
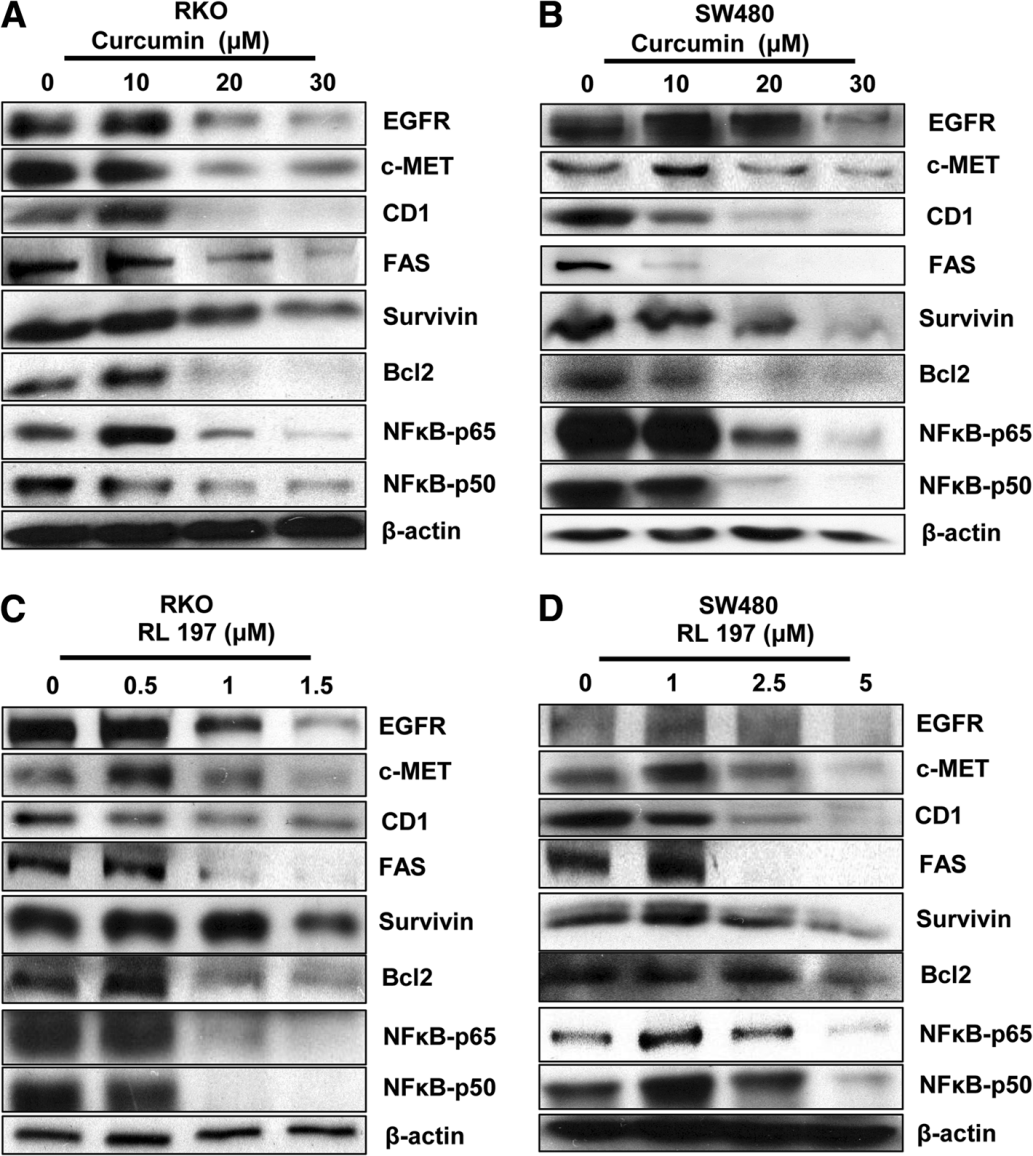
**Curcumin and RL197 decrease expression of Sp-regulated gene products.** Downregulation of Sp-regulated gene products by different concentration of curcumin in RKO (**A**) and SW480 (**B**) cells, and by different concentrations of RL197 in RKO (**C**) and SW480 (**D**) cells was determined by western blot analysis as described in the Materials and Methods. Experiments with Sp proteins (Figure 2) and Sp-regulated gene products were part of the same study and have the same β-actin loading controls.

Curcumin induces a broad spectrum of anticancer activities in multiple cancer cell lines; however, the low bioavailability of this compound has spurred interest in development of different structural classes of analogs including the heterocyclic and cyclohexanone derivatives investigated in this study [13,14]. Figure 1 illustrates the structures of these compounds and their respective IC_50_ values (half-maximal) for inhibition of RKO colon cancer cell growth. The IC_50_ value for curcumin was 10 μM and values for the 10 analogs ranged from 0.7 to 4.8 μM, with the RL197 analog being the most potent compound. In contrast, non-transformed CCD-18Co colon cells were relatively resistant to the growth inhibitory effects of RL197 and <30% growth inhibition was observed in cells treated with 10 μM RL197 (Additional file 1: Figure S1A) Figures 2A and 2B illustrate the concentration-dependent inhibition of RKO and SW480 colon cancer cell growth after treatment with curcumin and RL197, and comparable effects were observed in both cell lines. Previous studies in this laboratory show that the anticancer activity of curcumin is due, in part, to downregulation of Sp1, Sp3, Sp4 and pro-oncogenic Sp-regulated genes [20,21], and results in Figures 2C and 2D confirm that curcumin and RL197 also decrease expression of Sp1, Sp3 and Sp4 proteins in RKO and SW480 cells. We also examined the effects of curcumin (Figures 3A and 3B) and RL197 (Figures 3C and 3D) on expression of several Sp-regulated genes in RKO and SW480 cells, respectively. Both compounds decreased levels of gene products that play roles in cell proliferation (EGFR, cMET and cyclin D1), survival (survivin and bcl-2), inflammation (p65 and p50), and metabolism/drug transport (fatty acid synthase (FAS)). In addition, we also observed that curcumin and RL197 increased Annexin V staining (a marker of apoptosis) in RKO and SW480 cells (Additional file 1: Figures S1B and 1C). These results are consistent with the effects of curcumin in other cell lines [20,21] where Sp transcription factors are an important target for this drug.

### Curcumin and RL197 activate ROS in colon cancer cells

**Figure 4:**
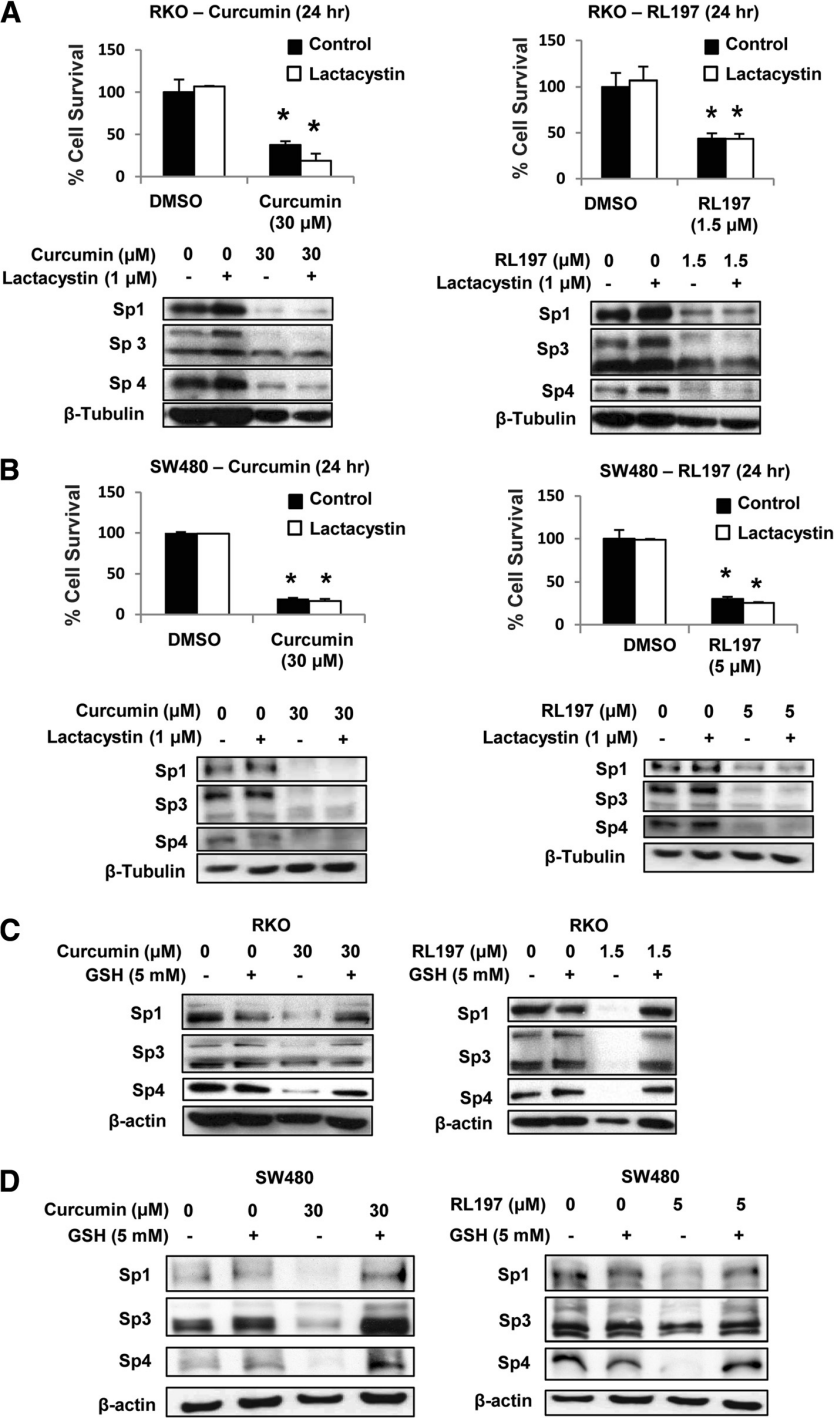
**Curcumin and RL197-mediated activation of proteasomes and ROS.** RKO (**A**) and SW480 (**B**) cells were treated with DMSO (control), curcumin, or RL197 alone or in combination with 1 μM lactacystin for 24 hr ,and effects on cell proliferation or expression of Sp1, Sp3 or Sp4 proteins (western blots) were determined as outlined in the Materials and Methods. RKO (**C**) and SW480 (**D**) cells were treated with DMSO, curcumin, RL197 alone or in combination with 5 mM GSH for 24 hr, and whole cell lysates were analyzed by western blot analysis as described in the Materials and Methods. Cell proliferation results (A, B) are means ± S.E. for at least 3 replicate experiments, and significant (p < 0.05) growth inhibition is indicated (*).

Drug-induced downregulation of Sp1, Sp3 and Sp4 is cell context- and compound-dependent and involves activation of proteasomes or ROS [20,21,23,29,33-38]. Results summarized in Figures 4A and 4B demonstrate that curcumin- and RL197-mediated effects on Sp1, Sp3 and Sp4 were not affected after cotreatment with the proteasome inhibitor lactacystin in RKO and SW480 cells, respectively. Moreover, in parallel experiments, lactacystin did not affect curcumin- or RL197-mediated growth inhibition. Figures 4C and 4D show that curcumin- and RL197-mediated downregulation of Sp transcription factors in RKO and SW480 cells, respectively, was attenuated after cotreatment with the antioxidant GSH. These results are comparable to those observed for curcumin in pancreatic cancer cells [21], suggesting that curcumin- and RL197-dependent effects on Sp1, Sp3 and Sp4 are ROS-dependent.

**Figure 5:**
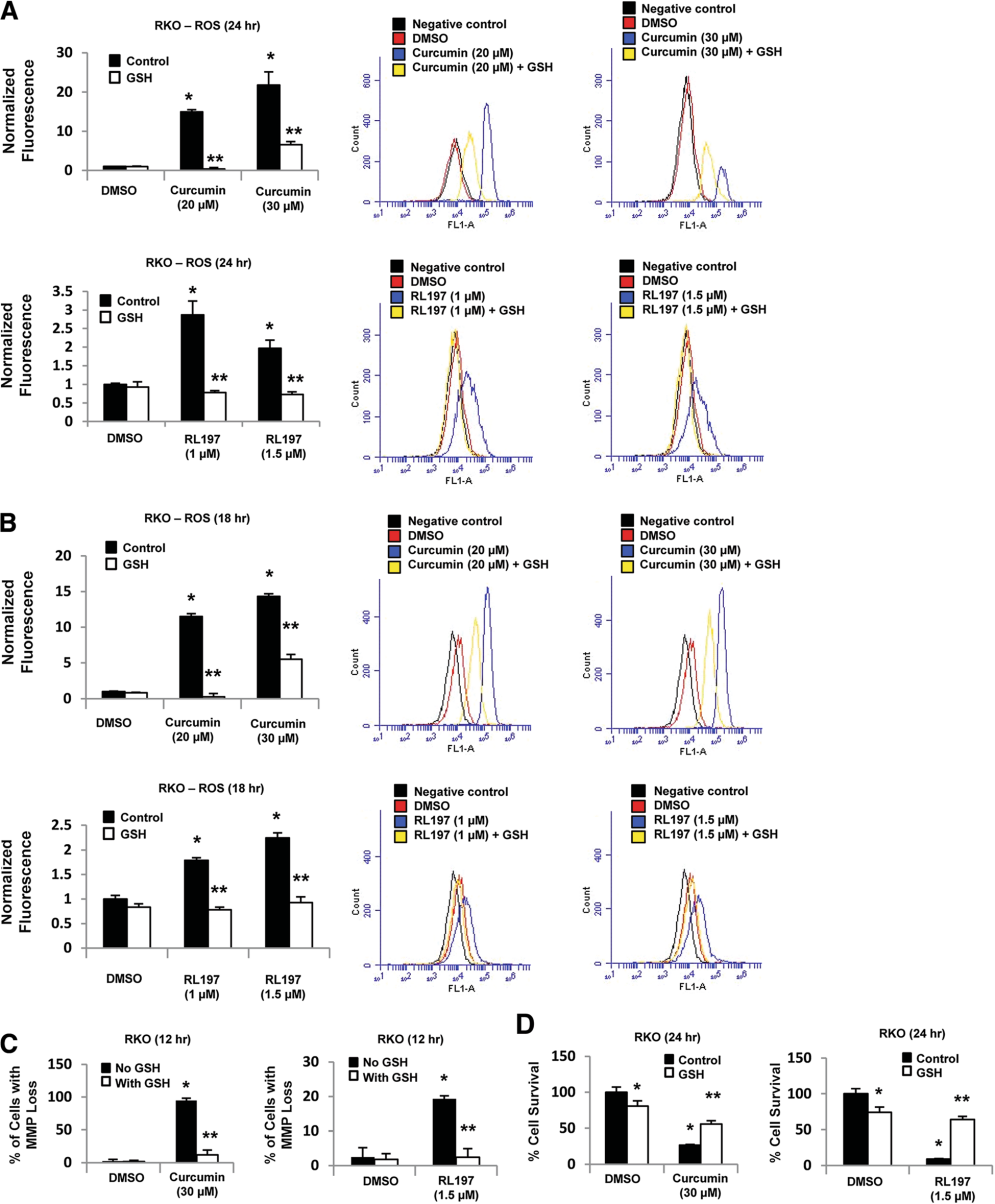
**The antioxidant GSH attenuates curcumin and RL197-induced responses.** RKO cells were treated with curcumin and RL197 for 24 (**A**) or 18 (**B**) hr, and induction of ROS was investigated by FACS analysis as outlined in the Materials and Methods. RKO cells were treated with DMSO, curcumin, and RL197 alone and in combination with GSH for 12 (**C**) or 24 (**D**) hr, and effects on MMP or cell proliferation, respectively, were determined as outlined in the Materials and Methods. Results are means ± S.E. for at least 3 replicate determinations, and significant (p < 0.05) induction of ROS, MMP or cell proliferation (*) and inhibition after cotreatment with GSH (**) is indicated.

RKO cells were used as a model to investigate induction of ROS and its role in mediating the effects of curcumin and RL197. Figures 5A and 5B show that both curcumin and RL197 significantly induced ROS formation after treatment for 24 and 18 hr, respectively, and this response was attenuated after cotreatment with the antioxidant GSH. The magnitude of ROS induction by curcumin was much higher than observed for RL197, and induction of ROS by RL197 was minimal at all time points ≤ 18 hr. In contrast, curcumin significantly induced ROS in RKO cells after treatment for 6, 12, 18 and 24 hr (Additional file 2: Figure S2). Curcumin and RL197 decreased MMP in RKO cells and this response was also attenuated after cotreatment with GSH (Figure 5C). These results suggest that RL197 directly effects mitochondria resulting in ROS production, whereas the parallel induction of ROS and decreased MMP observed after treatment with curcumin is consistent with a more complex mechanism of ROS induction involving extramitochondrial pathways, and this is currently being investigated. Despite differences in the magnitude and mechanisms of ROS induction by curcumin and RL197, their effects on RKO cell growth inhibition were also inhibited after cotreatment with GSH (Figure 5D), confirming the critical role of ROS in mediating the cytotoxicity of curcumin and RL197 in colon cancer cells. We also observed that 5 mM GSH alone slightly inhibited RKO cell proliferation; however, a <25% decrease in growth was observed (Figure 5D).

### Curcumin and RL197 disrupt miR27a:ZBTB10 and miR-20a/17-5p:ZBTB4 interactions

**Figure 6:**
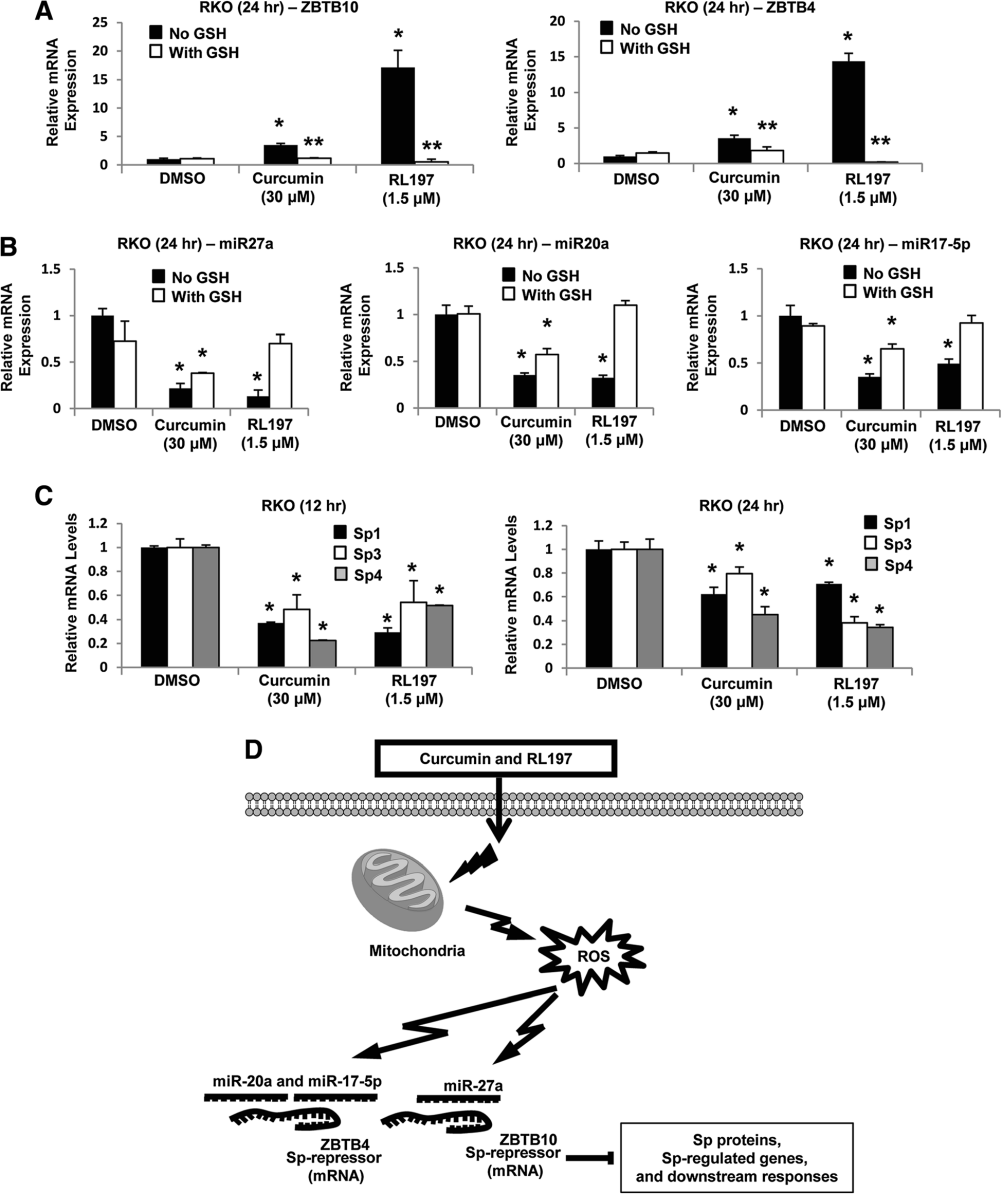
**Curcumin and RL197 disrupt miR-ZBTB10/ZBTB4 interactions.** RKO cells were treated with DMSO, curcumin, and RL197 alone or in combination with GSH, and induction of ZBTB10 or ZBTB4 (**A**) and downregulation of multiple miRs (**B**) were determined by real time PCR as outlined in the Materials and Methods. Results are means ± S.E. for at least 3 replicate determinations and significant (p < 0.05) induction of ZBTB10 or ZBTB4 and suppression of miRs (*) and inhibition of these responses by GSH (**) are indicated. (**C**) Curcumin and RL197-dependent downregulation of Sp1, Sp3 and Sp4 mRNA levels was determined in RKO cells as outlined in the Materials and Methods. Results are means ± S.E. (3 replicates) and significant (p < 0.05) inhibition is indicated. (**D**) Proposed mechanism of action of curcumin and RL197 in RKO cells.

ZBTB10 is a transcriptional “Sp repressor” regulated by miR-27a in cancer cell lines, and previous studies show that drug-induced Sp repression is due to ROS-dependent disruption of miR-27a:ZBTB10 [29,34,38]. Treatment of RKO cells with curcumin or RL197 induced expression of ZBTB10 and this was attenuated after cotreatment with GSH. A recent report identified ZBTB4 as a second “Sp repressor” [26], and we also observed that curcumin and RL197 induced ZBTB4 expression and this was partially inhibited after cotreatment with GSH (Figure 6A). Curcumin and RL197 decreased expression of miR-27a [25] and also miR-20a/miR-17-5p which regulate (inhibit) expression of ZBTB10 and ZBTB4, respectively [26], and these responses were also inhibited after cotreatment with GSH (Figure 6B). The induction of the transcriptional repressors ZBTB10 and ZBTB4 was accompanied by decreased expression of Sp1, Sp3 and Sp4 mRNA levels (Figure 6C) and their corresponding proteins (Figure 2). These results suggest a mechanism of action of curcumin and RL197 which is illustrated in Figure 6D. Induction of ROS by curcumin and RL197 results in ROS-dependent disruption of miR-ZBTB10 and miR-ZBTB4 and the subsequent induction of the transcriptional repressors results in suppression of genes with GC-rich promoters, including Sp1, Sp3, Sp4 and Sp-regulated genes.

## Discussion

Non-oncogene addiction by cancer cells is now recognized as an important pathway for maintaining the cancer cell phenotype [27,28] and Sp transcription factors are an example of non-oncogenes that fulfill this function. Sp1, Sp3 and Sp4 are overexpressed in multiple cancer cell lines and tumor types and Sp-regulated genes and oncogenes play an important role in cancer cell proliferation, survival, angiogenesis and inflammation [20-26,29-38]. These transcription factors are ideal for development of mechanism-based drugs since Sp1 expression decreases with age [39-41], and results of animal studies show that Sp1, Sp3 and Sp4 are highly expressed in tumor but not in non-tumor tissues [21,23]. The high expression of Sp transcription factors is due, in part, to miR-dependent repression of the Sp repressors ZBTB10 and ZBTB4, which competitively bind GC-rich gene promoters and deactivate transcription [25,26]. MiR-27a and miRs-20a/17-5p, which are overexpressed in many tumors interact with and suppress ZBTB10 or ZBTB4, respectively, and overexpression of ZBTB10 and ZBTB4 or transfection of cells with miR-27a and miR-20a/17-5p antagomirs also decrease expression of Sp transcription factors [25,26].

Several drugs that target Sp transcription factors have been identified and these include the non-steroidal anti-inflammatory drugs (NSAIDs) tolfenamic acid, COX-2 inhibitors and the nitro-NSAID GT-094, and several natural products including betulinic acid (BA), celastrol and the synthetic triterpenoids methyl 2-cyano-3,12-dioxooleana-a-dien-28-oate (CDDO-Me) and methyl 2-cyano-3,4-dioxo-18β-olean-1,12-dien-30-oate (CDODA-Me) [20-26,29-38]. In bladder cancer cells, curcumin induced proteasome-dependent downregulation of Sp1, Sp3 and Sp4 [20], whereas in pancreatic cancer cells this response was ROS-dependent and reversed by cotreatment with antioxidants such as GSH [21]. Both curcumin and the potent RL197 analog induced ROS (Figure 5A) and decreased expression of Sp1, Sp3, Sp4 and Sp-regulated proteins (Figures 2 and 3) in colon cancer cells, and cotreatment with GSH inhibited these responses and also partially reversed the growth inhibitory effects of these compounds (Figure 5D). Similar effects have previously been observed for BA and GT-094 in colon cancer cells and induction of ROS is also a critical element for their cytotoxicity [34,35]. However, the identities of individual ROS species induced by curcumin and RL197 have not been determined and are currently being investigated.

Increased ROS contributes to tumor formation due to several factors including oxidative DNA damage; however, several anticancer drugs also induce ROS and this plays an important role in their cancer chemotherapeutic activity [42,43]. Induction of ROS by CDDO-Me, BA/GT-094 and celastrol in pancreatic, colon and bladder cancer cells, respectively, results in downregulation of miR-27a and induction of ZBTB10 [29,34,35]. Moreover, celastrol also downregulates miR-20a and other miR paralogs with the same seed sequence in bladder cancer cells and this is accompanied by induction of ZBTB4. This study demonstrates that like celastrol, curcumin- and RL197-induced ROS in RKO cells also decreases miR-dependent regulation of ZBTB10 and ZBTB4 (Figure 6). Previous studies show that curcumin induces ROS in some cancer cell lines [44-48], and results of this study suggest that curcumin and RL197 induce ROS in RKO cells and ROS-mediated disruption of miR-ZBTB interactions results in downregulation of Sp transcription factors and Sp-regulated gene products. These results for curcumin and RL197 in colon cancer cells are consistent with previous studies with other ROS inducers which act as anticancer agents through the common pathway illustrated in Figure 6D.

## Conclusions

Previous reports show that the many anticancer agents such as curcumin and RL197 target Sp transcription factors and Sp-regulated genes [20-26,30-38] and thereby inhibit non-oncogene addiction by cancer cells and tumors. The mechanisms of Sp downregulation are both drug- and cell context-dependent, and this study demonstrates the important role of ROS in disrupting miR-mediated suppression of ZBTB10 and ZBTB4. Current studies are focused on the critical *trans*-acting factors that are induced or inhibited by ROS and are required to decrease miR-27a and miR-20a/17-5p expression and possibly other miRs that form part of the miR-23a~miR-27a~miR-24-2 and miR-17-92 clusters, respectively.

## Abbreviations

BA: Betulinic acid; CB: Cannabinoid; CDDO: 2-cyano-3,12-dioxoolean-1,9-dien-28-oic acid; CDODA: 2-cyano-3,11-dioxo-18β-olean-1,12-dien-30-oic acid; c-MET: Hepatocyte growth factor receptor; EGFR: Epidermal growth factor receptor; FAAH: Fatty acid amide hydrolase; ROS: Reactive oxygen species; siRNA: Small inhibitory RNA; Sp: Specificity protein; VEGF: Vascular endothelial growth factor; VEGFR: Vascular endothelial growth factor receptor.

## Competing interests

The authors declare they have no competing interests.

## Author’s contributions

SUG carried out the majority of the *in vitro* studies, analyzed and summarized the results, and helped draft this manuscript. KK co-supervised research and carried out PCR studies on miR expression. LL and RJR synthesized compounds and helped with drafting of this manuscript. SS conceived of this project, wrote the manuscript, and supervised research. All authors read and approved the final manuscript.

## Pre-publication history

The pre-publication history for this paper can be accessed here:

http://www.biomedcentral.com/1471-2407/12/564/prepub

## Supplementary Material

Additional file 1 **Figure S1.** Effects of RL197 on proliferation of CCD-18Co colon fibroblasts (A) and induction of Annexin V staining by curcumin and RL197 in RKO (B) and SW480 (C) colon cancer cells. Click here for file

Additional file 2 **Figure S2.** ROS induction by curcumin. RKO cells were treated with 20 or 30 μM curcumin alone or with GSH. ROS was determined 6, 12, 18 or 24 hr after treatment by FACS analysis as outlined in the Materials and Methods. Click here for file
