# Supplementary material for: Curcumin and synthetic analogs induce reactive oxygen species and decreases specificity protein (Sp) transcription factors by targeting microRNAs
Source: BMC Cancer. 2012 Nov 30;12:564. doi: 10.1186/1471-2407-12-564 (PMC3522018; doi:10.1186/1471-2407-12-564)
Supplement: Additional file 1 — Figure S1. Effects of RL197 on proliferation of CCD-18Co colon fibroblasts (A) and induction of Annexin V staining by curcumin and RL197 in RKO (B) and SW480 (C) colon cancer cells. [file 1471-2407-12-564-S1.pdf]

Supplemental Figure 1

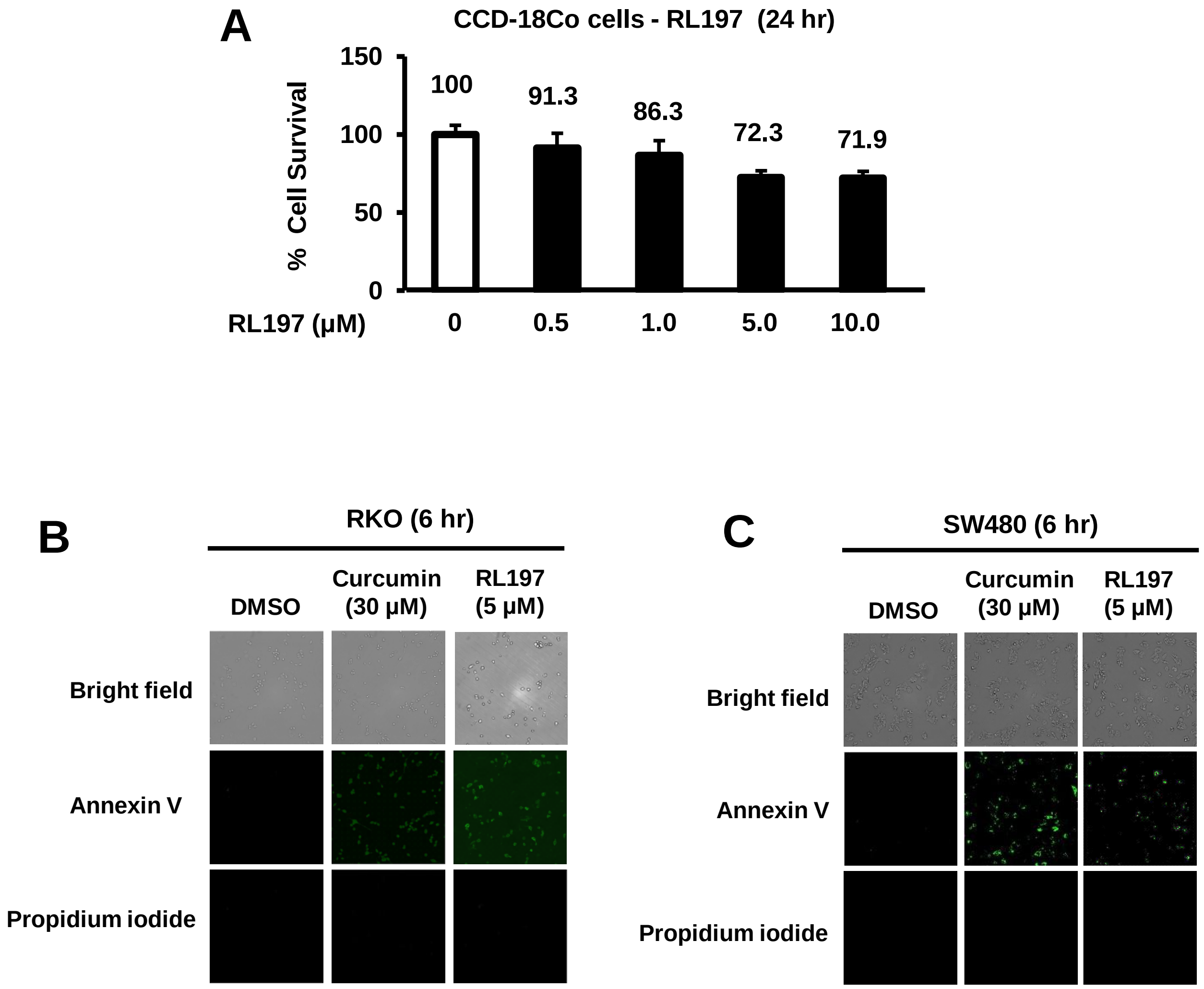

Supplemental Figure 1. Effects of RL197 on proliferation of CCD-18Co colon fibroblasts (A) and induction of Annexin V staining by curcumin and RL197 in RKO (B) and SW480 (C) colon cancer cells.
