# Supplementary material for: Curcumin and synthetic analogs induce reactive oxygen species and decreases specificity protein (Sp) transcription factors by targeting microRNAs
Source: BMC Cancer. 2012 Nov 30;12:564. doi: 10.1186/1471-2407-12-564 (PMC3522018; doi:10.1186/1471-2407-12-564)
Supplement: Additional file 2 — Figure S2. ROS induction by curcumin. RKO cells were treated with 20 or 30 μM curcumin alone or with GSH. ROS was determined 6, 12, 18 or 24 hr after treatment by FACS analysis as outlined in the Materials and Methods. [file 1471-2407-12-564-S2.pdf]

Supplemental Figure 2

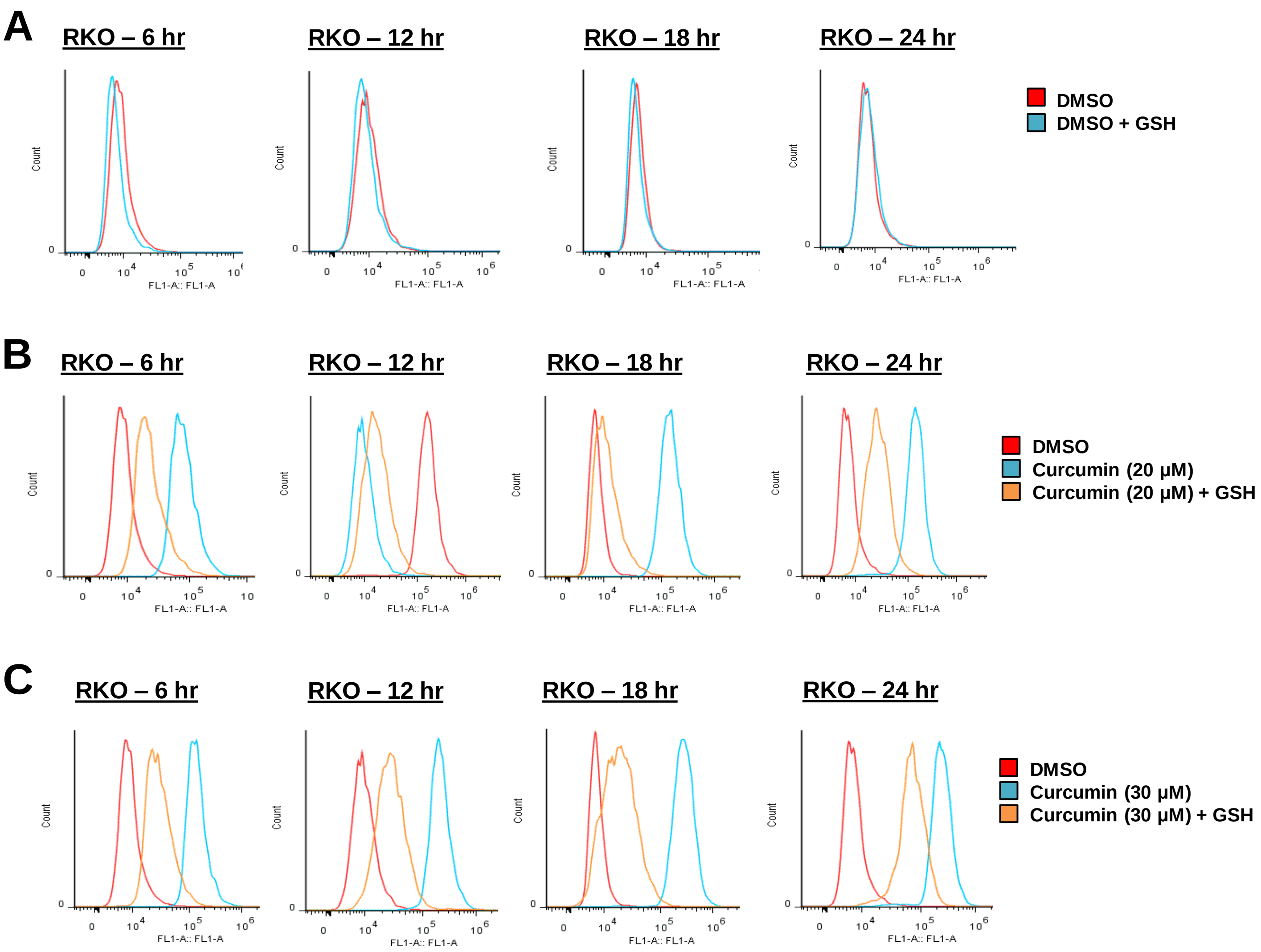

Supplemental Figure 2. ROS induction by curcumin. RKO cells were treated with 20 or 30 μM curcumin alone or with GSH. ROS was determined 6, 12, 18 or 24 hr after treatment by FACS analysis as outlined in the Materials and Methods.
